# Supplementary material for: Distinct Expression Patterns of VEGFR 1-3 in Gastroenteropancreatic Neuroendocrine Neoplasms: Supporting Clinical Relevance, but not a Prognostic Factor
Source: J Clin Med. 2020 Oct 21;9(10):3368. doi: 10.3390/jcm9103368 (PMC7589439; doi:10.3390/jcm9103368)
Supplement: Supplementary file 1 [file jcm-09-03368-s001.pdf]

Supplementary table 1. Overview of the four grades of immunohistochemical staining for VEGFR 1-3.

Receptor	Grading	n	%
VEGFR 1	Negative	39	15.7
	Weakly positive	63	25.3
	Moderately positive	90	36.1
	Strongly positive	57	22.9
VEGFR 2	Negative	189	75.9
	Weakly positive	44	17.7
	Moderately positive	15	6.0
	Strongly positive	1	0.4
VEGFR 3	Negative	24	9.6
	Weakly positive	71	28.5
	Moderately positive	100	40.2
	Strongly positive	54	21.7
